# Influence of type 1 diabetes on basal and agonist‐induced permeability of the blood–brain barrier

**DOI:** 10.14814/phy2.12653

**Published:** 2015-12-10

**Authors:** William G. Mayhan, Jasmine P. Scott, Denise M. Arrick

**Affiliations:** ^1^ Department of Cellular Biology and AnatomyLSU Health Sciences Center‐ShreveportShreveportLouisiana

**Keywords:** Arterioles, endothelium, histamine, L‐NMMA, nitric oxide, venules

## Abstract

Type 1 diabetes mellitus (T1D) impairs endothelial nitric oxide synthase (eNOS)‐dependent responses of cerebral arterioles. However, the influence of T1D on another critical aspect of endothelial cell function in the cerebral microcirculation, i.e., regulation of permeability of the blood–brain barrier (BBB), remains largely unknown. Our goal was to examine basal and agonist‐induced changes in permeability of the BBB in nondiabetic and type 1 diabetic (streptozotocin; 50 mg/kg IP) rats. On the day of the experiment (2–3 months after streptozotocin), a craniotomy was made over the parietal cortex in nondiabetic and diabetic rats. We measured the permeability of the BBB (FITC‐dextran‐10K) under basal conditions and during application of histamine. We also measured diameter of cerebral arterioles in response to histamine in the absence and presence of NG‐monomethyl‐L‐arginine (L‐NMMA). We found that basal permeability of the BBB was elevated in T1D and application of histamine did not produce a further increase in permeability. In contrast, basal permeability of the BBB was minimal in nondiabetics and histamine produced an increase in permeability. In addition, histamine‐induced arteriolar dilation was less in diabetics than in nondiabetics, and vasodilation to histamine was inhibited by L‐NMMA. Our findings suggest that T1D‐induced endothelial dysfunction leads to an increase in basal permeability of the BBB, but decreases the ability of the endothelium of the BBB to respond to an important inflammatory mediator. Thus, T1D impairs two critical aspects of endothelial cell function in the cerebral microcirculation, i.e., basal and agonist‐induced changes in permeability of the BBB and arteriolar dilation.

## Introduction

Overwhelming evidence has shown that type 1 diabetes (T1D) impairs endothelial nitric oxide synthase (eNOS)‐ and neuronal nitric oxide synthase (nNOS)‐dependent dilation of cerebral arteries and arterioles via mechanisms that appear to favor oxidant‐producing over antioxidant‐protecting pathways (Mayhan 1989; Mayhan et al. 2006; Arrick et al. 2007b; Faraci 2011; Drummond and Sobey 2014). An equally critical role for the endothelium in the cerebral microcirculation, beyond vascular reactivity, involves the regulation of permeability of the blood–brain barrier (BBB). This barrier (at the level of arterioles, capillaries, and venules) is defined by a well‐established basement membrane, presence of tight junctions between adjacent endothelial cells, the absence of fenestrations, and a close proximity to other brain cell types, including astrocytes, pericytes, microglia, and neurons (Figueroa and Duling 2009; del Zoppo 2010; Chow and Gu 2015). These unique characteristics of this barrier relative to the typical peripheral endothelium confer highly restricted exchange of bloodborne molecules between the systemic circulation and the extracellular fluid compartment. The integrity of the BBB is determined by endothelium, pericytes, astrocytes and neurons, and the interaction of these structures (neurovascular unit) is termed neurovascular coupling (Hawkins and Davis 2005; Chow and Gu 2015). Alterations in neurovascular coupling, and hence the BBB, are associated with many serious abnormalities, including multiple sclerosis, Alzheimer's disease, cognitive impairment, epilepsy, and stroke (Hawkins and Davis 2005; Girouard and Iadecola 2006; Stanimirovic and Friedman 2012; Jing et al. 2013; Najjar et al. 2013; Joutel and Faraci 2014).

Although T1D produces abnormalities of the endothelium to impair vascular reactivity of cerebral arteries and arterioles, and disrupts the neurovascular unit/neurovascular coupling, to increase the risk of many disorders including cognition and stroke, there is a lack of information regarding the influence of T1D on other important aspects of endothelial cell function in the brain, i.e., regulation of permeability of the BBB. Although studies have shown that basal permeability of the BBB may be elevated in T1D (Oztas and Kucuk 1987; Knudsen and Jakobsen 1989; Hawkins et al. 2007) and that histamine increases the permeability of the BBB in animal models devoid of diabetes (Gross et al. 1982; Olesen 1987; Schilling and Wahl 1994; Sarker et al. 1998) via activation of several cellular pathways including the synthesis/release of nitric oxide (Baranczyk‐Kuzma et al. 1992; Joo 1993; Mayhan 1996), there is a void of studies that have examined the influence of T1D on histamine‐induced changes in permeability of the BBB. Thus, the first goal of this study was to examine the influence of T1D on basal permeability characteristics of the BBB. To accomplish this goal, we measured the clearance of fluorescein isothiocyanate dextran‐10,000 daltons (FITC‐dextran‐10K; molecular weight = 10,000 DA) from the pial microcirculation in nondiabetic and diabetic rats. Our second goal was to examine whether nitric oxide synthase‐dependent increases in permeability of the BBB are altered by T1D. To accomplish this goal, we examined the clearance of FITC‐dextran‐10K from the pial microcirculation in nondiabetic and diabetic rats during topical application of histamine. Our final goal was to determine the role of nitric oxide in reactivity of cerebral arterioles in response to histamine, and whether reactivity in response to histamine is altered by T1D.

## Materials and Methods

### Induction of diabetes

All procedures were reviewed and approved by the Institutional Animal Care and Use Committee at the Louisiana State University Health Sciences Center‐Shreveport. Male Sprague–Dawley rats (200–250 g body wt) were randomly divided into nondiabetic or diabetic groups. All rats had access to food and water ad libitum. The diabetic group of rats was injected with streptozotocin (50 mg/kg ip) to induce T1D and the nondiabetic group of rats was injected with vehicle (sodium citrate buffer). Blood samples, for measurement of blood glucose concentration, were obtained 3–5 days after injection of streptozotocin or vehicle, and on the day of the experiment (8–12 weeks later). An animal with a blood glucose concentration of greater than 300 mg/dL was considered to be diabetic.

### Preparation of animals

Rats were prepared for in vivo studies 8–12 weeks after injection of streptozotocin or vehicle. Rats were anesthetized (thiobutabarbital (Inactin); 100 mg/kg IP), and a tracheotomy was performed. The animals were ventilated and a cannula was inserted into a femoral vein for injection of the intravascular tracer, (FITC‐dextran‐10K; molecular weight = 10,000 DA) and supplemental anesthesia (10–30 mg/kg, if necessary). A femoral artery was cannulated for the measurement of arterial pressure, to obtain a blood sample for the measurement of blood glucose concentration and to obtain a blood sample for the determination of plasma concentration of FITC‐dextran‐10K.

After placement of all catheters, the animal was placed in a head holder and a craniotomy was made over the left parietal cortex (Mayhan and Heistad 1985). The cranial window was suffused with artificial cerebral spinal fluid and the temperature of the suffusate was maintained at 37 ± 1°C. The cranial window was connected via a three‐way valve to an infusion pump, which allowed for infusion of agonists (histamine or saline) into the suffusate. Arterial blood gases were monitored and maintained within normal limits.

### Permeability of the BBB

The permeability of the BBB was evaluated by calculating the clearance of FITC‐dextran‐10K (mL/sec × 10^−6^) as we have described previously (Mayhan and Heistad 1985; Mayhan 1996). In this study, we report the clearance of FITC‐dextran‐10K at various time points during a continuous application of vehicle (saline) or histamine over the cranial window preparation.

### Pial arteriolar diameter

Diameter of pial arterioles was measured using a video image‐shearing monitor. We measured the diameter of the largest pial arteriole exposed by the craniotomy before and during application of histamine (1.0 and 10 μmol/L) in the absence and presence of NG‐monomethyl‐L‐arginine (L‐NMMA) (10 μmol/L).

### Experimental protocol

In the first series of studies, we measured the basal permeability characteristics of the BBB in nondiabetic (*n* = 14) and diabetic (*n* = 9) rats. In these groups of animals, the cranial window was suffused for a control period of 30–45 min. Then, we started a continuous intravenous infusion of FITC‐dextran‐10K. Seventy‐two minutes after starting the infusion of FITC‐dextan‐10K, we applied vehicle (saline) to the cerebral microcirculation. Application of vehicle continued for the duration of the experiment (128 min).

In a second group of nondiabetic (*n* = 10) and diabetic (*n* = 11) rats, we examined the influence of topical application of histamine (10 μmol/L) on the permeability of the BBB. In these groups of rats, the cranial window was suffused for a control period of 30–45 min. Then, we started a continuous intravenous infusion of FITC‐dextran‐10K. Seventy‐two minutes after starting the infusion of FITC‐dextan‐10K, we applied histamine (10 μmol/L) to the cerebral microcirculation. Application of histamine continued for the duration of the experiment (128 min).

In a third group of nondiabetic (*n* = 11) and diabetic (*n* = 8) rats, we examined responses of cerebral arterioles to histamine (1 and 10 μmol/L) and nitroglycerin (1 and 10 μmol/L). In addition, in the nondiabetic rats, we determined the role of nitric oxide in histamine‐induced changes in pial arteriolar diameter by examining responses before and during a continuous topical application of L‐NMMA (10 μmol/L).

### Statistical analysis

Data are presented as means ± SE. Analysis of variance (ANOVA) with Fisher's test for significance was used to compare differences in clearance of FITC‐dextran‐10K at the various time points between the groups of animals. Baseline diameter of pial arterioles, responses of pial arterioles to histamine, body weight, blood glucose concentration, and mean arterial blood pressure between nondiabetic and diabetic rats were compared using unpaired *t*‐tests. A paired *t*‐test was used to compare responses of pial arterioles to histamine before and during application of L‐NMMA. A *P*‐value of 0.05 or less was considered to be significant.

## Results

### Baseline conditions

Mean arterial blood pressure and baseline diameter of cerebral arterioles were similar in nondiabetic and diabetic rats (Table 1). Blood glucose concentration was higher and body weight was lower in diabetic compared to nondiabetic rats (Table 1).

**Table 1:** Mean arterial pressure, baseline diameter of cerebral arterioles, blood glucose concentration and body weight in nondiabetic and diabetic rats

	Nondiabetic	Diabetic
Mean arterial pressure (mmHg)	107 ± 4	109 ± 7
Baseline diameter (microns)	44 ± 2	46 ± 3
Blood glucose (mg/dL)	117 ± 3	506 ± 25^a^
Body weight (grams)	356 ± 11	288 ± 17^a^

Values are means ± SE.

a *P* < 0.05 versus nondiabetic rats.

### Basal permeability of BBB

**Figure 1:**
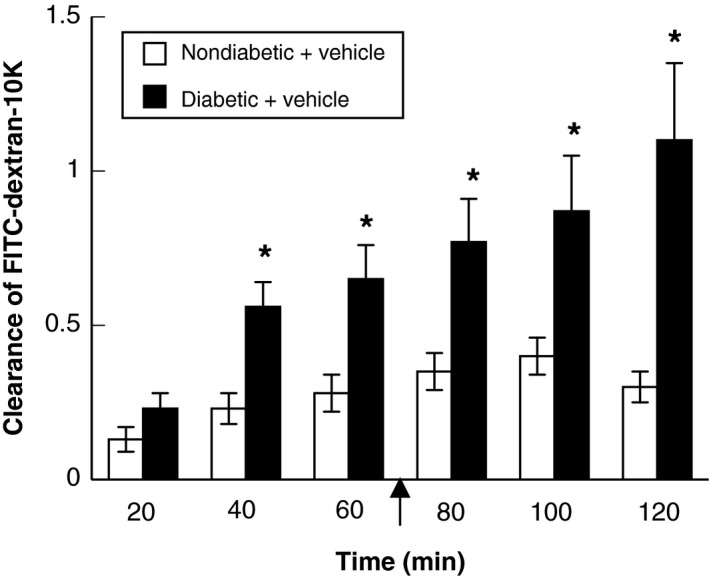
Clearance of FITC‐dextran‐10K at various time intervals in nondiabetic (open bars) and diabetic (closed bars) rats under basal conditions. Intravenous infusion of FITC‐dextran‐10K was started at time 0 (not shown) and continued for the duration of the experimental protocol. Arrow indicates starting the superfusion with vehicle (saline; time = 72 min after starting intravenous infusion of FITC‐dextran‐10K). Values are means ± SE. *P <* *0.05 versus diabetic rats.

In nondiabetic rats, clearance of FITC‐dextran‐10K over the experimental time frame remained modest (Fig. 1). In contrast, clearance of FITC‐dextran‐10K in diabetic rats steadily increased over the experimental period, and was greater than that observed in nondiabetic rats at various time points during the experimental period (Fig. 1). Thus, basal permeability of the BBB is increased by T1D.

### Permeability of the BBB in response to histamine

**Figure 2:**
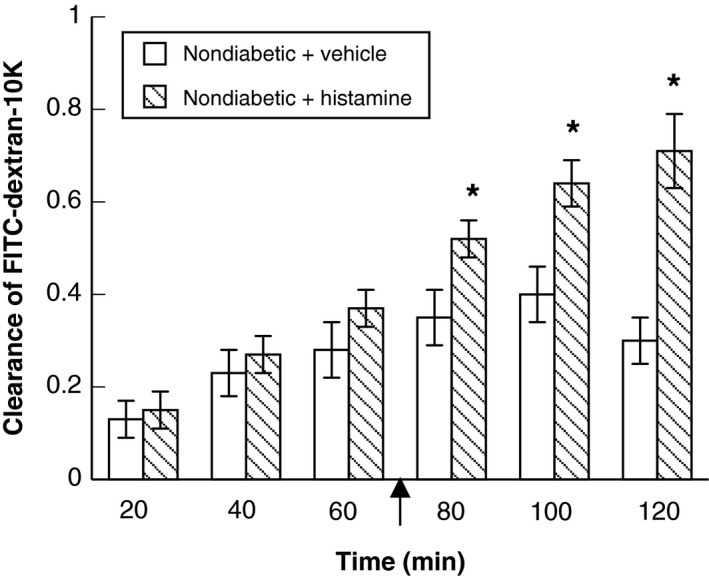
Clearance of FITC‐dextran‐10K in nondiabetic rats during superfusion with vehicle (saline) (open bars) or histamine (hatched bars). Intravenous infusion of FITC‐dextran‐10K was started at time 0 (not shown) and continued for the duration of the experimental protocol. Arrow indicates starting the superfusion with histamine (10 μmol/L; time = 72 min after starting intravenous infusion of FITC‐dextran‐10K). Values are means ± SE. *P <* *0.05 versus response during superfusion with vehicle.

**Figure 3:**
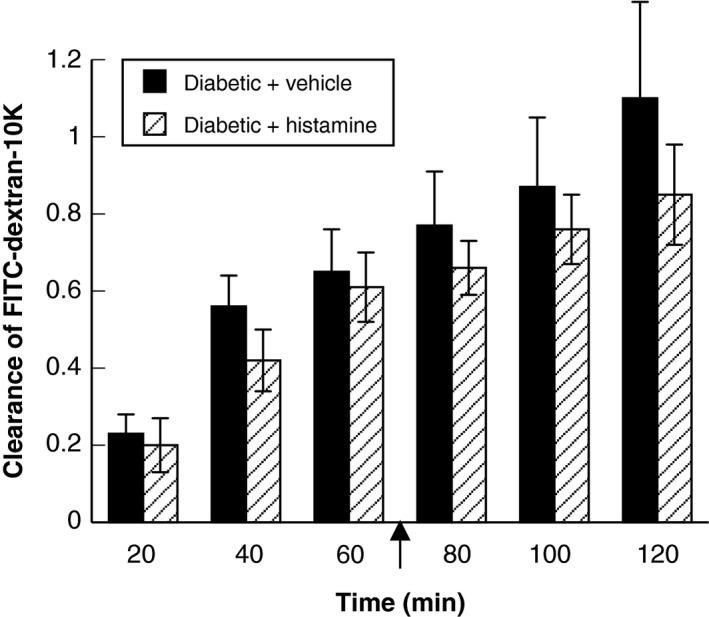
Clearance of FITC‐dextran‐10K in diabetic rats during superfusion with vehicle (saline) (closed bars) or histamine (hatched bars). Intravenous infusion of FITC‐dextran‐10K was started at time 0 (not shown) and continued for the duration of the experimental protocol. Arrow indicates starting the superfusion with histamine (10 μmol/L; time = 72 min after starting intravenous infusion of FITC‐dextran‐10K). Values are means ± SE.

Histamine produced an increase in the clearance of FITC‐dextran‐10K from the cerebral microcirculation in nondiabetic rats (Fig. 2), and the clearance of FITC‐dextran‐10K remained elevated at all time points during the superfusion with histamine when compared with superfusion with vehicle (Fig. 2). In contrast, superfusion with histamine in diabetic rats did not increase the clearance of FITC‐dextran‐10K from the cerebral microcirculation above that seen with superfusion with vehicle (Fig. 3). Thus, it appears that T1D impairs histamine‐induced increases in permeability of the BBB.

### Responses of cerebral arterioles

**Figure 4:**
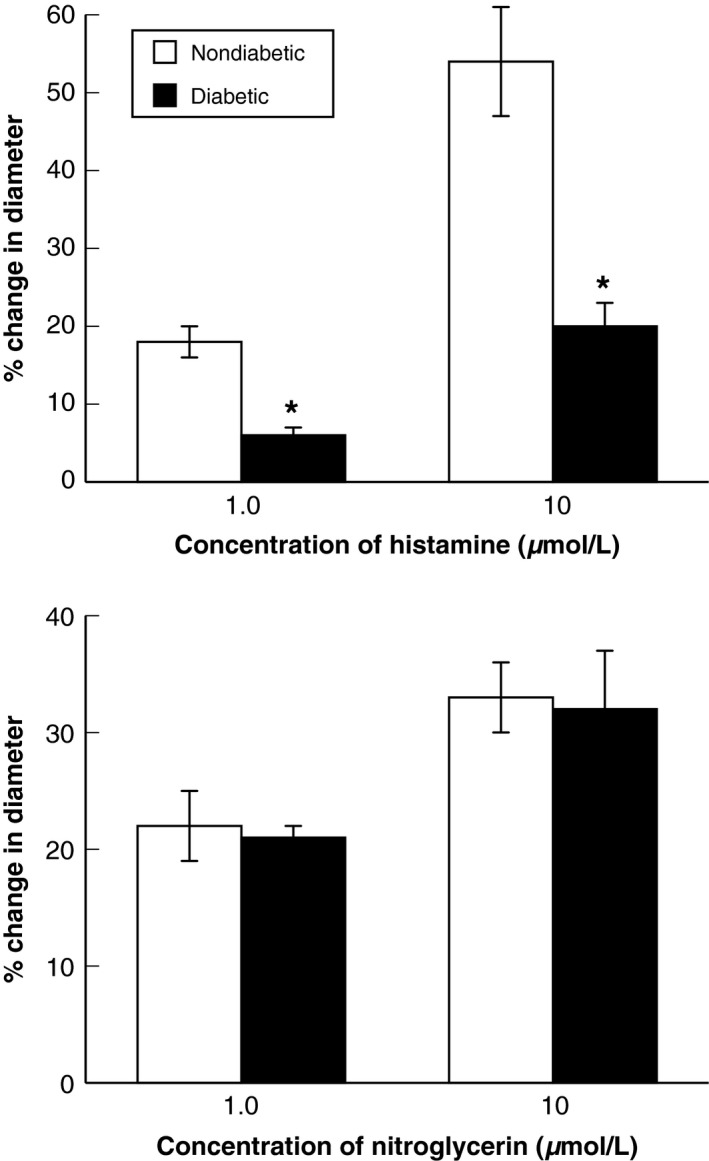
Responses of cerebral arterioles to histamine and nitroglycerin in nondiabetic (open bars) and diabetic (closed bars) rats. Values are means ± SE. *P <* *0.05 versus diabetic rats.

Histamine and nitroglycerin produced dose‐related dilation of cerebral arterioles in nondiabetic and diabetic rats (Fig. 4). However, the magnitude of vasodilation in response to histamine was less in diabetic compared to nondiabetic rats. Histamine (1 and 10 μmol/L) dilated cerebral arterioles by 18 ± 2% and 53 ± 7%, respectively in nondiabetic rats, but by only 6 ± 1% and 20 ± 3%, respectively in diabetic rats. In contrast, nitroglycerin produced similar dose‐related dilation of cerebral arterioles in nondiabetic and diabetic rats (Fig. 4). Topical application of L‐NMMA (10 μmol/L) to the cerebral microcirculation produced a small, but significant constriction of cerebral arterioles in nondiabetic rats (9 ± 1%). Dilation of cerebral arterioles in response to histamine (1.0 μmol/L) was significantly decreased by L‐NMMA (18 ± 1% before vs. 3 ± 1% during L‐NMMA), while responses to nitroglycerin were not altered by L‐NMMA (23 ± 3% before vs. 23 ± 2% during L‐NMMA). Thus, dilation of cerebral arterioles in response to histamine appears to be related to the synthesis/release of nitric oxide, presumably via activation of eNOS, and T1D impairs responses of cerebral arterioles to histamine.

## Discussion

There are four findings from this study. First, there was an increase in basal permeability of the BBB in T1D rats compared to nondiabetics. Second, superfusion of the cerebral microcirculation with histamine produced an increase in permeability of the BBB in nondiabetic, but not in diabetic rats. Third, dilation of cerebral arterioles in response to histamine was less in diabetic versus nondiabetic rats. Fourth, dilation of cerebral arterioles in response to histamine was dependent, in part, upon the synthesis/release of nitric oxide. These findings suggest that T1D can influence two critical aspects of endothelial cell function during physiologic and pathophysiologic conditions, i.e., reactivity of cerebral arterioles and permeability of the BBB.

### Permeability of the BBB

We used a well‐established methodology to evaluate basal and histamine‐induced changes in permeability of the BBB (Mayhan and Heistad 1985, 1986; Mayhan 1996). No leakage of FITC‐dextran‐10K was observed from dura, skin, or bone during suffusion with vehicle or histamine, and we suggest that the clearance of FITC‐dextran‐10K represents alterations in permeability that occur in cerebral microvessels exposed by the craniotomy in nondiabetic and diabetic rats.

Several studies have examined the effects of T1D on basal permeability of the BBB, but the findings have been mixed. A few investigators have shown that short‐term (2 weeks) and long‐term (>4 weeks) T1D did not produce any discernible increase in basal permeability of the BBB to large and small molecules (Jakobsen et al. 1978; Ennis and Betz 1986; Knudsen et al. 1986; Lorenzi et al. 1986). Others, however, have shown that short‐term and long‐term T1D increases basal permeability of the BBB to large and small molecules (Stauber et al. 1981; Oztas and Kucuk 1987; Huber et al. 2006; Reeson et al. 2015). Using the cranial window technique, we also (Mayhan 1990) found that the clearance of FITC‐albumin from the cerebral microcirculation and the number of venular leaky sites were modestly elevated in diabetic (3–5 months) when compared to nondiabetic rats. The discrepancy between studies regarding an influence of T1D on basal permeability of the BBB is not entirely clear, but the diversity in the findings certainly emphasizes the need for more studies to examine the regional characteristics of the BBB during health and disease. In this study, we found that the permeability of the BBB to a low‐molecular weight tracer (FITC‐dextran‐10K) was elevated in diabetic rats. The cellular pathway, i.e., transcellular and/or paracellular, by which T1D can influence the basal permeability of the BBB is not entirely clear from this study. However, others have shown that tight junctional proteins (occludin and ZO‐1) are decreased in experimental models of diabetes (Chehade et al. 2002; Hawkins et al. 2007). Thus, it is likely that a breakdown in tight junctional proteins could account for the increase in basal permeability of the BBB. Given that there appears to be a correlation between increased permeability of the BBB and neurological deficits (Starr et al. 2003), and that T1D appears to produce an increase in basal permeability of the BBB, it is conceivable that the increase in permeability could contribute to the pathogenesis of many CNS‐associated diseases in which an increase in BBB permeability is observed, including stroke, Alzheimer's disease, epilepsy, and multiple sclerosis. In any event, our findings suggest that T1D produces a profound and diverse influence on an important aspect of endothelial cell function in the brain, i.e., regulation of basal permeability of the BBB.

We also examined whether histamine could produce an increase in permeability of the BBB in nondiabetic and diabetic rats. The rationale for these studies is based upon previous studies that have shown that T1D impairs eNOS‐dependent dilation of cerebral arterioles to important vasoactive agonists, including histamine (Ayajiki et al. 1992; Arrick et al. 2007a). Given that the primary site of disruption of the BBB is in venules and veins (Mayhan and Heistad 1985) and that nitric oxide is important in histamine‐induced changes in permeability of the BBB (Mayhan 1996), we wondered whether T1D could impair these important aspects of endothelial cell function that involved both arterioles (reactivity) and venules (permeability). Many investigators have examined the influence of histamine on the BBB, but most of these studies have concentrated on an examination of the BBB in control animal models, and few, if any, have examined the permeability of the BBB during T1D. For the most part, studies have shown that stimulation with histamine produces an opening of the BBB in cell culture models and in animal models (Gross et al. 1982; Olesen 1987; Butt and Jones 1992; Revest et al. 1994; Schilling and Wahl 1994). In addition, it appears that histamine acts via both H1 and H2 receptors to induce changes in permeability of the BBB via activation of cAMP (Joo et al. 1975; Schilling and Wahl 1994). The cellular route for changes in permeability of the BBB in response to histamine is not entirely clear. Although early studies using peripheral blood vessels have shown that histamine produces an increase in permeability via a paracellular route (contraction of adjacent endothelial cells) (Majno and Palade 1961a,b), others (Joo et al. 1975) have speculated that as histamine may activate cAMP in cerebral vessels, changes in permeability in response to histamine may occur via a transcellular (vesicular) route. In this study, we found that topical application of histamine produced an increase in permeability of the BBB in nondiabetic rats, but did not alter the permeability of the BBB in diabetic rats. Using our in vivo approach, we are not able to determine the route by which histamine increased the permeability of the BBB in nondiabetic rats. Our finding that histamine did not increase the permeability of the BBB in T1D was a bit surprising given that others (Yuan et al. 2014) have shown that the permeability characteristics of peripheral blood vessels in response to inflammatory mediators are greater in T1D. As basal permeability was increased, and presumably pathways that contributed to this increase would be more susceptible in T1D, we considered the possibility that histamine would produce a greater increase in permeability in diabetic than in nondiabetic rats. However, this was not the case. There are several possible explanations for this finding. First, it may relate to differences between vascular beds, i.e., responses in the mesentery during T1D may be different from that for the brain. Second, a teleological explanation might relate to the brain being enclosed in rigid structure, and further change in permeability in T1D in response to inflammatory mediators would be extremely detrimental to brain function. Third, it is conceivable that the increase in basal permeability observed in diabetic rats may be related to elevated levels of histamine in the plasma and/or brain tissue as reported by others (Hollis et al. 1985; Gill et al. 1988, 1989), and further stimulation with exogenous histamine would not produce an additional change in permeability. Fourth, it is conceivable that impairment in nitric oxide synthase‐dependent mechanisms and/or elevations in oxidative stress during T1D impair the ability of the cerebral endothelium to respond to histamine and produce a further increase in permeability of the BBB. Fifth, it is conceivable that changes in permeability in response to histamine may not be directed at the endothelium, but may involve other cellular elements and that T1D impairs the ability of these cells to regulate the permeability of the BBB. Evidences suggests that T1D also produces alterations in microglia, neurons, and astrocytes (Mukai et al. 1980; Revsin et al. 2005; Currais et al. 2012; Jing et al. 2013; Hu et al. 2015), and as the cell types may also influence permeability of the BBB (Abbott et al. 2010), it is possible that alterations in these cell types may inhibit/prevent agonist‐induced changes in permeability of the BBB. Thus, although the precise mechanism is not clear, our findings suggest that histamine‐induced changes in permeability of the BBB are less in T1D.

### Reactivity of cerebral arterioles

To determine whether T1D impaired another important aspect of endothelial cell function, we examined responses of cerebral arterioles to topical application of histamine in nondiabetic and diabetic rats. We also examined the role of nitric oxide in histamine‐induced dilation of cerebral arterioles by testing response to histamine before and after treatment with L‐NMMA. First, we found that T1D impaired dilation of cerebral arterioles in response to histamine, but not to nitroglycerin. Several studies, including studies from our laboratory, have shown that eNOS‐ and nNOS‐dependent responses of cerebral arterioles are impaired by T1D via mechanisms that appear to favor oxidant‐producing over antioxidant‐protecting pathways (Mayhan 1989; Mayhan et al. 2006; Arrick et al. 2007a,b; Faraci 2011; Drummond and Sobey 2014). In addition, it appears that histamine dilates cerebral arterioles via the synthesis/release of nitric oxide, presumably via activation of eNOS (Ayajiki et al. 1992; Karagiannis et al. 2003; Arrick et al. 2007a). Thus, it appears that T1D produces selective impairment of endothelium‐dependent responses in cerebral arterioles.

In summary, to our knowledge, this is the first study to examine the effects of T1D on two important aspects of endothelial cell function in cerebral venules and arterioles, i.e., regulation of basal and agonist‐induced changes in permeability of the BBB, and regulation of vasodilation. We found that T1D increased basal permeability of the BBB, but impaired the ability of histamine to increase the permeability of the BBB. We also found that histamine‐induced dilation of cerebral arterioles was dependent upon the synthesis/release of nitric oxide and that T1D impaired histamine‐induced dilation of cerebral arterioles. Taken together, these findings suggest that T1D impairs two critical aspects of endothelial cell function in cerebral venules and arterioles, i.e., regulation of basal and agonist‐induced changes in permeability of the BBB, and regulation of vasodilation. Given that there appears to be a correlation between alterations in permeability of the BBB and neurological deficits, it is conceivable that the increases in basal permeability and/or lack of ability to respond to agonists that increase the permeability of the BBB may have important implications to the pathogenesis of many CNS‐associated diseases, including stroke, Alzheimer's disease, epilepsy, and multiple sclerosis.

## Conflict of Interest

None declared.

## References

[phy212653-bib-0001] Abbott, N. J. , A. A. Patabendige , D. E. Dolman , S. R. Yusof , and D. J. Begley . 2010 Structure and function of the blood‐brain barrier. Neurobiol. Dis. 37:13–25. 19664713 10.1016/j.nbd.2009.07.030

[phy212653-bib-0002] Arrick, D. M. , G. M. Sharpe , H. Sun , and W. G. Mayhan . 2007a Diabetes‐induced cerebrovascular dysfunction: role of poly(ADP‐ribose) polymerase. Microvasc. Res. 73:1–6. 16982071 10.1016/j.mvr.2006.08.001

[phy212653-bib-0003] Arrick, D. M. , G. M. Sharpe , H. Sun , and W. G. Mayhan . 2007b nNOS‐dependent reactivity of cerebral arterioles in Type 1 diabetes. Brain Res. 1184:365–371. 17991456 10.1016/j.brainres.2007.10.004 PMC2174607

[phy212653-bib-0004] Ayajiki, K. , T. Okamura , and N. Toda . 1992 Involvement of nitric oxide in endothelium‐dependent, phasic relaxation caused by histamine in monkey cerebral arteries. Jpn. J. Pharmacol. 60:357–362. 1287271 10.1254/jjp.60.357

[phy212653-bib-0005] Baranczyk‐Kuzma, A. , K. L. Audus , F. L. Guillot , and R. T. Borchardt . 1992 Effects of selected vasoactive substances on adenylate cyclase activity in brain, isolated brain microvessels, and primary cultures of brain microvessel endothelial cells. Neurochem. Res. 17:209–214. 1311435 10.1007/BF00966802

[phy212653-bib-0006] Butt, A. M. , and H. C. Jones . 1992 Effect of histamine and antagonists on electrical resistance across the blood‐brain barrier in rat brain‐surface microvessels. Brain Res. 569:100–105. 1611469 10.1016/0006-8993(92)90374-i

[phy212653-bib-0007] Chehade, J. M. , M. J. Haas , and A. D. Mooradian . 2002 Diabetes‐related changes in rat cerebral occludin and zonula occludens‐1 (ZO‐1) expression. Neurochem. Res. 27:249–252. 11958524 10.1023/a:1014892706696

[phy212653-bib-0008] Chow, B. W. , and C. Gu . 2015 The molecular constituents of the blood‐brain barrier. Trends Neurosci. 38:598–608. 26442694 10.1016/j.tins.2015.08.003 PMC4597316

[phy212653-bib-0009] Currais, A. , M. Prior , D. Lo , C. Jolivalt , D. Schubert , and P. Maher . 2012 Diabetes exacerbates amyloid and neurovascular pathology in aging‐accelerated mice. Aging Cell 11:1017–1026. 22938075 10.1111/acel.12002 PMC3500443

[phy212653-bib-0010] Drummond, G. R. , and C. G. Sobey . 2014 Endothelial NADPH oxidases: which NOX to target in vascular disease. Trends Endocrinol. Metab. 25:452–463. 25066192 10.1016/j.tem.2014.06.012

[phy212653-bib-0011] Ennis, S. R. , and A. L. Betz . 1986 Sucrose permeability of the blood‐retinal and blood‐brain barriers. Invest. Ophthalmol. Vis. Sci. 27:1095–1102. 3721787

[phy212653-bib-0012] Faraci, F. M. 2011 Protecting against vascular disease in brain. Am. J. Physiol. Heart Circ. Physiol. 300:H1566–H1582. 21335467 10.1152/ajpheart.01310.2010 PMC3094081

[phy212653-bib-0013] Figueroa, X. F. , and B. R. Duling . 2009 Gap junctions in the control of vascular function. Antioxid. Redox Signal. 11:251–266. 18831678 10.1089/ars.2008.2117 PMC2933153

[phy212653-bib-0014] Gill, D. S. , C. S. Thompson , and P. Dandona . 1988 Increased histamine in plasma and tissues in diabetic rats. Diabetes Res. 7:31–34. 3402164

[phy212653-bib-0015] Gill, D. S. , M. A. Barradas , V. A. Fonseca , and P. Dandona . 1989 Plasma histamine concentrations are elevated in patients with diabetes mellitus and peripheral vascular disease. Metabolism 38:243–247. 2918844 10.1016/0026-0495(89)90082-6

[phy212653-bib-0016] Girouard, H. , and C. Iadecola . 2006 Neurovascular coupling in the normal brain and in hypertension, stroke, and Alzheimer disease. J. Appl. Physiol. 100:328–335. 16357086 10.1152/japplphysiol.00966.2005

[phy212653-bib-0017] Gross, P. M. , G. M. Teasdale , D. I. Graham , W. J. Angerson , and A. M. Harper . 1982 Intra‐arterial histamine increases blood‐brain barrier transport in rats. Am. J. Physiol. 243:H307–H317. 7114240 10.1152/ajpheart.1982.243.2.H307

[phy212653-bib-0018] Hawkins, B. T. , and T. P. Davis . 2005 The blood‐brain barrier/neurovascular unit in health and disease. Pharmacol. Rev. 57:173–185. 15914466 10.1124/pr.57.2.4

[phy212653-bib-0019] Hawkins, B. T. , T. F. Lundeen , K. M. Norwood , H. L. Brooks , and R. D. Egleton . 2007 Increased blood‐brain barrier permeability and altered tight junctions in experimental diabetes in the rat: contribution of hyperglycaemia and matrix metalloproteinases. Diabetologia 50:202–211. 17143608 10.1007/s00125-006-0485-z

[phy212653-bib-0020] Hollis, T. M. , J. A. Kern , N. A. Enea , and A. J. Cosgarea . 1985 Changes in plasma histamine concentration in the streptozotocin‐diabetic rat. Exp. Mol. Pathol. 43:90–96. 3159596 10.1016/0014-4800(85)90058-9

[phy212653-bib-0021] Hu, P. , J. S. Thinschmidt , S. Caballero , S. Adamson , L. Cole , T. Chan‐Ling , et al. 2015 Loss of survival factors and activation of inflammatory cascades in brain sympathetic centers in type 1 diabetic mice. Am. J. Physiol. Endocrinol. Metab. 308:E688–E698. 25714673 10.1152/ajpendo.00504.2014 PMC4398829

[phy212653-bib-0022] Huber, J. D. , R. L. VanGilder , and K. A. Houser . 2006 Streptozotocin‐induced diabetes progressively increases blood‐brain barrier permeability in specific brain regions in rats. Am. J. Physiol. Heart Circ. Physiol. 291:H2660–H2668. 16951046 10.1152/ajpheart.00489.2006

[phy212653-bib-0023] Jakobsen, J. , L. Malmgren , and Y. Olsson . 1978 Permeability of the blood‐nerve barrier in the streptozotocin diabetic rat. Exp. Neurol. 60:277–285. 207549 10.1016/0014-4886(78)90083-3

[phy212653-bib-0024] Jing, Y. H. , K. H. Chen , P. C. Kuo , C. C. Pao , and J. K. Chen . 2013 Neurodegeneration in streptozotocin‐induced diabetic rats is attenuated by treatment with resveratrol. Neuroendocrinology 98:116–127. 23486084 10.1159/000350435

[phy212653-bib-0025] Joo, F. 1993 The role of second messenger molecules in the regulation of permeability in the cerebral endothelial cells. Adv. Exp. Med. Biol. 331:155–164. 8333330 10.1007/978-1-4615-2920-0_25

[phy212653-bib-0026] Joo, F. , Z. Rakonczay , and M. Wollemann . 1975 CAMP mediated regulation of the permeability of brain capillaries. Experientia 31:582–584. 237773 10.1007/BF01932471

[phy212653-bib-0027] Joutel, A. , and F. M. Faraci . 2014 Cerebral small vessel disease: insights and opportunities from mouse models of collagen IV‐related small vessel disease and cerebral autosomal dominant arteriopathy with subcortical infarcts and leukoencephalopathy. Stroke 45:1215–1221. 24503668 10.1161/STROKEAHA.113.002878 PMC3966958

[phy212653-bib-0028] Karagiannis, J. , J. J. Reid , I. Darby , P. Roche , M. J. Rand , and C. G. Li . 2003 Impaired nitric oxide function in the basilar artery of the obese Zucker rat. J. Cardiovasc. Pharmacol. 42:497–505. 14508235 10.1097/00005344-200310000-00007

[phy212653-bib-0029] Knudsen, G. M. , and J. Jakobsen . 1989 Blood‐brain barrier permeability to sodium. modification by glucose or insulin? J. Neurochem. 52:174–178. 2642296 10.1111/j.1471-4159.1989.tb10913.x

[phy212653-bib-0030] Knudsen, G. M. , J. Jakobsen , M. Juhler , and O. B. Paulson . 1986 Decreased blood‐brain barrier permeability to sodium in early experimental diabetes. Diabetes 35:1371–1373. 3770313 10.2337/diab.35.12.1371

[phy212653-bib-0031] Lorenzi, M. , D. P. Healy , R. Hawkins , J. M. Printz , and M. P. Printz . 1986 Studies on the permeability of the blood‐brain barrier in experimental diabetes. Diabetologia 29:58–62. 2937677 10.1007/BF02427282

[phy212653-bib-0032] Majno, G. , and G. E. Palade . 1961a Studies on inflammation I. The effect of histamine and serotonin on vascular permeability: an electron microscopic study. J. Biophys. Biochem. Cytol. 11:571–605. 14468626 10.1083/jcb.11.3.571 PMC2225138

[phy212653-bib-0033] Majno, G. , and G. E. Palade . 1961b Studies on inflammation II. The site of action of histamine and serotonin along the vascular tree: a topographic study. J. Biophys. Biochem. Cytol. 11:607–626. 14468625 10.1083/jcb.11.3.607 PMC2225127

[phy212653-bib-0034] Mayhan, W. G. 1989 Impairment of endothelium‐dependent dilatation of cerebral arterioles during diabetes mellitus. Am. J. Physiol. 256:H621–H625. 2923230 10.1152/ajpheart.1989.256.3.H621

[phy212653-bib-0035] Mayhan, W. G. 1990 Effect of diabetes mellitus on disruption of the blood‐brain barrier during acute hypertension. Brain Res. 534:106–110. 2073577 10.1016/0006-8993(90)90118-u

[phy212653-bib-0036] Mayhan, W. G. 1996 Role of nitric oxide in histamine induced increases in permeability of the blood‐brain barrier. Brain Res. 743:70–76. 9017232 10.1016/s0006-8993(96)01021-9

[phy212653-bib-0037] Mayhan, W. G. , and D. D. Heistad . 1985 Permeability of blood‐brain barrier to various sized molecules. Am. J. Physiol. 248:H712–H718. 2581459 10.1152/ajpheart.1985.248.5.H712

[phy212653-bib-0038] Mayhan, W. G. , and D. D. Heistad . 1986 Role of veins and cerebral venous pressure in disruption of the blood‐brain barrier. Circ. Res. 59:216–220. 3742745 10.1161/01.res.59.2.216

[phy212653-bib-0039] Mayhan, W. G. , D. M. Arrick , G. M. Sharpe , K. P. Patel , and H. Sun . 2006 Inhibition of NAD(P)H oxidase alleviates impaired NOS‐dependent responses of pial arterioles in Type 1 diabetes mellitus. Microcirculation 13:567–575. 16990215 10.1080/10739680600885194

[phy212653-bib-0040] Mukai, N. , S. Hori , and M. Pomeroy . 1980 Cerebral lesions in rats with streptozotocin‐induced diabetes. Acta Neuropathol. 51:79–84. 7435144 10.1007/BF00688853

[phy212653-bib-0041] Najjar, S. , D. M. Pearlman , O. Devinsky , A. Najjar , and D. Zagzag . 2013 Neurovascular unit dysfunction with blood‐brain barrier hyperpermeability contributes to major depressive disorder: a review of clinical and experimental evidence. J. Neuroinflammation 10:142. 24289502 10.1186/1742-2094-10-142 PMC4220803

[phy212653-bib-0042] Olesen, S. P. 1987 Leakiness of rat brain microvessels to fluorescent probes following craniotomy. Acta Physiol. Scand. 130:63–68. 3109211 10.1111/j.1748-1716.1987.tb08112.x

[phy212653-bib-0043] Oztas, B. , and M. Kucuk . 1987 Blood‐brain barrier permeability in streptozotocin‐induced diabetic rats. Med. Sci. Res. 15:645–646.

[phy212653-bib-0044] Reeson, P. , K. A. Tennant , K. Gerrow , J. Wang , S. Weiser Novak , K. Thompson , et al. 2015 Delayed inhibition of VEGF signaling after stroke attenuates blood‐brain barrier breakdown and improves functional recovery in a comorbidity‐dependent manner. J. Neurosci. 35:5128–5143. 25834040 10.1523/JNEUROSCI.2810-14.2015 PMC6705411

[phy212653-bib-0045] Revest, P. A. , H. C. Jones , and N. J. Abbott . 1994 Transendothelial electrical potential across pial vessels in anaesthetized rats: a study of ion permeability and transport at the blood‐brain barrier. Brain Res. 652:76–82. 7525022 10.1016/0006-8993(94)90319-0

[phy212653-bib-0046] Revsin, Y. , F. Saravia , P. Roig , A. Lima , E. R. de Kloet , F. Homo‐Delarche , et al. 2005 Neuronal and astroglial alterations in the hippocampus of a mouse model for type 1 diabetes. Brain Res. 1038:22–31. 15748869 10.1016/j.brainres.2004.12.032

[phy212653-bib-0047] Sarker, M. H. , A. S. Easton , and P. A. Fraser . 1998 Regulation of cerebral microvascular permeability by histamine in the anaesthetized rat. J. Physiol. 507(Pt 3):909–918. 9508849 10.1111/j.1469-7793.1998.909bs.x PMC2230814

[phy212653-bib-0048] Schilling, L. , and M. Wahl . 1994 Opening of the blood‐brain barrier during cortical superfusion with histamine. Brain Res. 653:289–296. 7982064 10.1016/0006-8993(94)90403-0

[phy212653-bib-0049] Stanimirovic, D. B. , and A. Friedman . 2012 Pathophysiology of the neurovascular unit: disease cause or consequence? J. Cereb. Blood Flow Metab. 32:1207–1221. 22395208 10.1038/jcbfm.2012.25 PMC3390807

[phy212653-bib-0050] Starr, J. M. , J. Wardlaw , K. Ferguson , A. MacLullich , I. J. Deary , and I. Marshall . 2003 Increased blood‐brain barrier permeability in type II diabetes demonstrated by gadolinium magnetic resonance imaging. J. Neurol. Neurosurg. Psychiatry 74:70–76. 12486269 10.1136/jnnp.74.1.70 PMC1738177

[phy212653-bib-0051] Stauber, W. T. , S. H. Ong , and R. S. McCuskey . 1981 Selective extravascular escape of albumin into the cerebral cortex of the diabetic rat. Diabetes 30:500–503. 7014313 10.2337/diab.30.6.500

[phy212653-bib-0052] Yuan, D. , S. Xu , and P. He . 2014 Enhanced permeability responses to inflammation in streptozotocin‐induced diabetic rat venules: rho‐mediated alterations of actin cytoskeleton and VE‐cadherin. Am. J. Physiol. Heart Circ. Physiol. 307:H44–H53. 24778164 10.1152/ajpheart.00929.2013 PMC4080175

[phy212653-bib-0053] del Zoppo, G. J. 2010 The neurovascular unit in the setting of stroke. J. Intern. Med. 267:156–171. 20175864 10.1111/j.1365-2796.2009.02199.x PMC3001328

